# Band-Like Brainstem Lesion in a Patient With a History of Lung Adenocarcinoma

**DOI:** 10.7759/cureus.30726

**Published:** 2022-10-26

**Authors:** Koji Obara

**Affiliations:** 1 Neurology, National Hospital Organization Akita National Hospital, Yurihonjo, JPN

**Keywords:** mri, lung adenocarcinoma, leptomeningeal metastasis, egfr, brainstem

## Abstract

Leptomeningeal metastasis (LM) is a severe complication of primary malignancy that has spread to the leptomeninges and cerebrospinal fluid (CSF). Here, we report a patient whose magnetic resonance imaging (MRI) showed a unique brainstem lesion suspicious of LM. A 72-year-old man presented with dizziness, gait instability, and cognitive decline, primarily object naming. He had a history of lung adenocarcinoma with epidermal growth factor receptor (EGFR) mutation. Brain MRI revealed a band-like lesion surrounding the ventral brainstem with T2 weighted-image/fluid attenuation inversion recovery (FLAIR) imaging and diffusion-weighted imaging (DWI) hyperintensity without gadolinium enhancement. No malignant cells were detected in the CSF. He underwent ventriculoperitoneal shunt two months after the onset, and his gait improved, but his cognitive function declined further. Recent reports suggest similar brainstem lesions as a unique LM pattern, which occurs almost exclusively in patients with lung adenocarcinoma with EGFR mutation. Therefore, if MRI shows this brainstem finding, repeated and appropriate CSF cytology is needed to detect tumor cells. Furthermore, if a patient with lung adenocarcinoma shows a cognitive decline, cerebral LM and auto-antibodies that mainly target neuronal surface antigens should be considered.

## Introduction

Leptomeningeal metastasis (LM) is a severe complication of primary malignancy that has spread to the leptomeninges and cerebrospinal fluid (CSF) [1,2]. Lung cancer is one of the most common neoplasms causing LM, and the condition has a poor prognosis if untreated [1,2]. Therefore, early detection of LM may contribute to neurological function preservation and improved life expectancy [2]. The diagnostic gold standard for LM is CSF cytology and contrast-enhanced T1 weighted image (T1WI) on magnetic resonance imaging (MRI) using gadolinium [3]. Typically, LM is revealed as focal or diffuse, nodular or linear enhancement of the leptomeninges along the brain sulci and cistern on contrast-enhanced T1WI [4-7]. However, some recent case reports describe a band-like lesion surrounding the ventral brainstem with restricted diffusion and without gadolinium enhancement on MRI as a unique LM pattern [5,6,8-12]. Moreover, this LM pattern occurs almost exclusively in patients with lung adenocarcinoma with epidermal growth factor receptor (EGFR) mutation [6,8-12]. Herein, we report a patient with a history of lung adenocarcinoma with EGFR mutation who presented with a band-like ventral brainstem lesion on MRI and simultaneously showed a rapid cognitive decline.

## Case presentation

**Figure 1:**
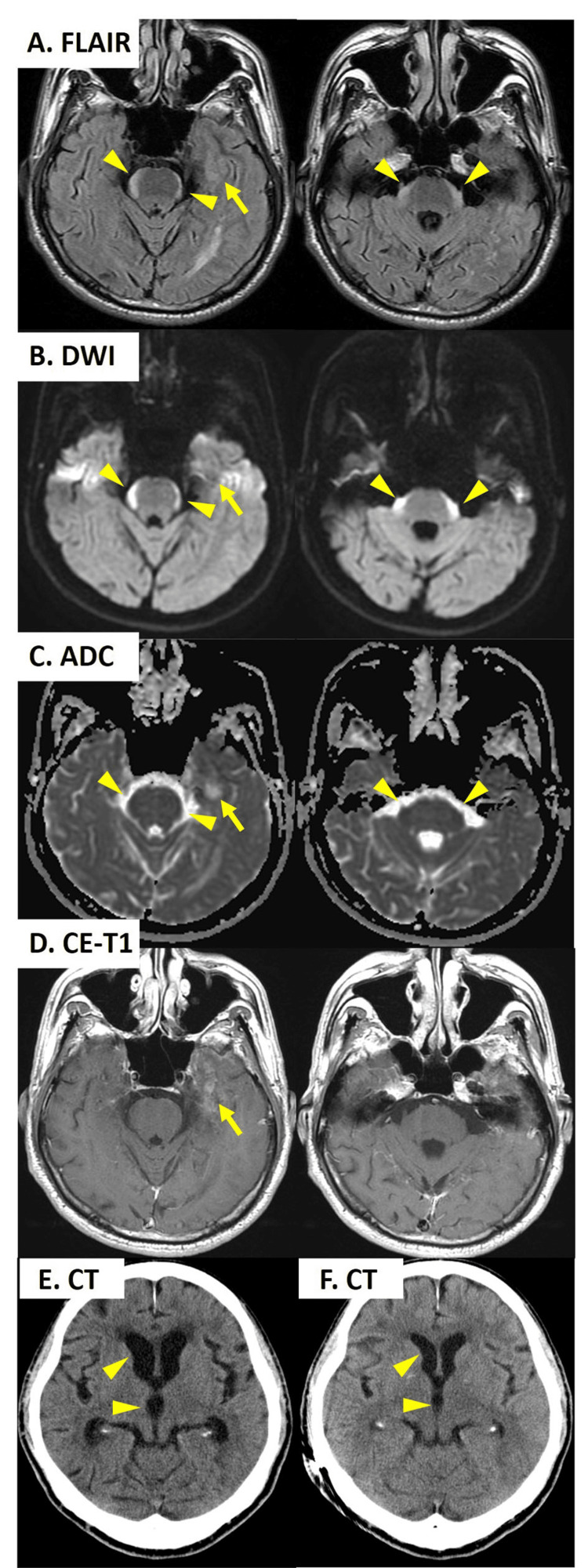
Brain magnetic resonance imaging (MRI) findings. (A) Fluid attenuation inversion recovery (FLAIR) imaging shows a band-like high-intensity lesion surrounding the ventral brainstem (arrowheads) and a slight high-intensity lesion in the left inner temporal lobe (arrow). (B) Diffusion-weighted imaging (DWI). (C) Apparent diffusion coefficient (ADC) mapping. The brainstem lesion shows a restricted pattern of the high intensity on DWI and low on ADC (arrowheads). Conversely, the left temporal lesion shows an unrestricted diffusion pattern (arrow). (D) Contrast-enhanced T1 weighted image (CE-T1). The brainstem lesion is not enhanced with the gadolinium contrast agent while the left inner temporal lesion is slightly enhanced (arrow). (E, F) Head computed tomography (CT) findings. (E) CT shows mild ventriculomegaly of the lateral and third ventricles before the ventriculoperitoneal shunt (arrowheads). (F) After the shunt, the ventriculomegaly improves (arrowheads).

A 72-year-old non-smoker male patient with a medical history of hypertension and hyperlipidemia visited our institute with a monthly record of progressive dizziness and gait instability. According to his wife, he became emotionally flat and indifferent to his surroundings around the same time. He had lost 3 kg in the last month. He underwent a thoracoscopic surgery for adenocarcinoma of the upper lobe of the right lung at stage IA according to International Union against Cancer classification (7th edition) with EGFR mutation (exon 21 L858R substitution) six years ago. On neurological examination, he was cooperative but repeated the same question. He spoke fluently but had difficulty naming objects. Cranial nerves appeared intact except for bilateral gaze-evoked horizontal nystagmus. The Romberg sign was positive. Simple gait was wide-based. One-foot standing and tandem gait was unable to perform. According to the Medical Research Council score, the muscle strength in his upper and lower limbs was 5/5. The finger-nose-finger test and the heel-shin test revealed mild oscillation and dysmetria without laterality. There was no sensory impairment. The tendon reflexes of the four extremities were symmetrically hyperactive and bilateral Babinski signs were elicited. He did not have nuchal rigidity. He scored 24/30 on Mini-Mental State Examination as he lost points for the name of the place, registration, and recall. In laboratory studies, total blood count and blood biochemistry including vitamin B1 and B12 were unremarkable. Serum tumor marker revealed elevated levels of CEA of 7.1 ng/mL and normal levels of SLX, proGRP, and NSE. The serum paraneoplastic neurological syndrome antibody panel returned negative including anti-Hu, anti-Ri, anti-amphiphysin, anti-CV2, anti-Ma2/Ta, anti-recoverin, anti-SOX1, anti-zic4, anti-GAD65, anti-titin and anti-Tr. The opening pressure on the lumber puncture was 15 cmH_2_O. A CSF study disclosed slight lymphocytic pleocytosis with 20 leukocytes/mm^3^ including 82% lymphocytes and a protein count of 1.4 g/L. The glucose level was 44 mg/dL with a CSF-to-serum glucose ratio of 40%. No malignant cells were seen on microscopy. The infectious work-up in CSF was negative. Brain MRI showed a slight enhancing lesion in the left inner temporal lobe with a gadolinium contrast agent, suggesting parenchymal metastasis (Figure 1A). Additionally, there was a band-like lesion surrounding the ventral brainstem with a high signal on fluid attenuation inversion recovery (FLAIR) imaging and a restricted diffusion pattern on diffusion-weighted imaging (DWI) and apparent diffusion coefficient (ADC) mapping (Figures 1B, 1C). There was no contrast enhancement in this brainstem lesion. The lateral and third ventricles presented mild hydrocephalus on head computed tomography (Figures 1D-1F).

**Figure 2:**
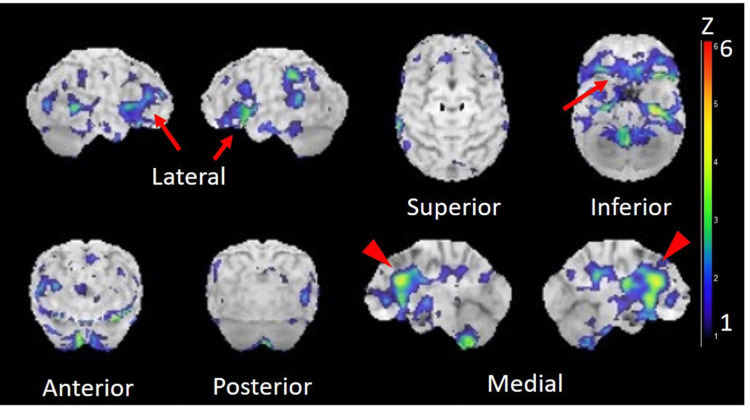
SPECT showing hypoperfusion in cortices Cerebral blood flow single-photon emission computed tomography using N-isopropyl-p-[123I] iodoamphetamine (IMP-SPECT) reveals hypoperfusion in cortices including the bilateral inferior frontal, orbital (arrows) and anterior cingulate gyri (arrowhead). The Z-score increases as the degree of the decrease in cerebral blood flow are larger than that of an age-matched normal database.

Single-photon emission CT using N-isopropyl-p-[123I] iodoamphetamine (IMP-SPECT) revealed hypoperfusion in cortices including the bilateral inferior frontal, orbital and anterior cingulate gyri (Figure 2).

Thoracic, abdominal, and pelvic computed tomography with contrast agent did not reveal any lesions suggesting metastasis. Positron emission tomography (PET) scan was not performed. His difficulty in walking and cognitive function deteriorated further. Two months after the onset, he underwent ventriculoperitoneal shunt. A CSF study was also performed at that time, but no malignant cells were detected. The family refused a brain biopsy during this shunt surgery. After the shunt surgery, his ataxic gait almost completely resolved. However, his object naming further deteriorated, and he could no longer speak. In contrast, he maintained his civility and was calm. Four months after onset, he died suddenly at home. The family refused post-mortem examination.

## Discussion

We found two important clinical points. First, in our patient with a history of lung adenocarcinoma, a brain MRI revealed a band-like lesion surrounding the ventral brainstem with T2WI/FLAIR and DWI hyperintensity and without gadolinium enhancement. We reviewed the English literature in PubMed and identified 22 cases with brainstem MRI findings that were similar to that of our patient (Table 1) [5,6,8-12].

**Table 1:** Previous reports of a band-like lesion surrounding the ventral brainstem with restricted diffusion and without gadolinium enhancement CT, chemotherapy; EGFR, epidermal growth factor receptor; Ig, immunoglobulin; IV, intravenous; LA, lung adenocarcinoma; LM, leptomeningeal metastasis; N/A, not available; TKI, thyroxine kinase inhibitor.

Reference	Number of Cases (Age/Sex)	Primary cancer	Systemic metastasis	Malignant cells in cerebrospinal fluid	EGFR mutation	Chemotherapy before LM	Therapy for LM	Prognosis
Brooks et al. [5]	1 (86F)	LA	Possible lung-to-lung	Negative	N/A	None	None	Deceased (10 weeks)
Khil et al. [6]	2 (75M, 47F)	2/2 LA	Bone, brain	Positive	1 suggested positive, 1 N/A	Case1 Gefitinib. Case 2 Docetaxel + carboplatin.	N/A	Case 1 Deceased (1 month). Case 2 Hospice care
Mitsuya et al. [8]	11 (mean 60, M1F10)	11/11 LA	9/11 brain	10/11positive	10/11 positive	11/11 Systemic chemotheraoy (10 EGFR-TKI)	Shunt surgery and whole brain radiotherapy in the representive case	Deterioration in the representive case
Cheng et al. [9]	4 (39-81, M2, F2)	4/4 LA	4 lung-to-lung, 2 brain, 2 bone	1 positive, 1 negative, 2 N/A	4/4 positive	2 Gefitinib plus. 1 Erlotinib plus. 1 None.	Case 1 IV steroid, Ig, Intrathecal CT, Erlonitib. Case 2 Brain radiotherapy. Case 3 Gefitinib, Afatinib. Case 4 Brain radiotherapy, Osimertinib.	Case 1 Deceased (1 year). Case 2 Lost to follow-up. Case 3 Deceased (7 months). Case 4 Survived over 6 months.
Crombe et al. [10]	1 (56M)	LA	Bone, lung-to-lung	Positive	Positive	Gefinitib	N/A	N/A
Maeda et al. [11]	2 (55M, 77M)	2/2 LA	N/A	2/2 positive	1 positive, 1 N/A	N/A	N/A	N/A
Yokota et al. [12]	1 (60F)	LA	Bone	Inconclusive	Positive	Erlotinib	Erlotinib	Improved MRI findings
	22	22/22 LA	18/21 systemic metastais	15/20 positive	17 positive, 1 suggested positive, 1 negative, 3 N/A	16 EGFR-TKI		
Our case	72M	LA	Possible brain	Negative	Positive	None	Shunt surgery	Deceased (4 months)

All cases had a lung adenocarcinoma or a history of lung adenocarcinoma [5,6,8-12]. Seventeen cases had EGFR mutations [6,8-12]. Eighteen cases concurrently suffered from the brain, bone, and lung-to-lung metastasis and had undergone systemic chemotherapy or radiotherapy [5,6,8-10,12]. Malignant cells in CSF were identified in 15 cases and were diagnosed as LM due to lung adenocarcinoma [6,8-12]. Although these cases had a poor prognosis in general, some cases that were treated with whole-brain radiotherapy and tyrosine kinase inhibitors for the brain lesion had relatively longer survival [5,6,8-12].

LM occurs in approximately 5% of non-small cell lung cancers, including adenocarcinoma [4]. Contrast-enhanced T1WI is usually the most sensitive MRI sequence to detect LM and usually shows a focal or diffuse, nodular, or linear enhancement of the leptomeninges along the brain sulci and cisterns [4-7]. In contrast, the band-like lesions surrounding the ventral brainstem in all cases including our patient were nonenhancing [5,6,8-12]. It is not fully understood why these band-like lesions are not enhanced. According to chronological observation in mouse models and clinical cases, it has been shown that early LM may be initially impermeable to contrast agents and nonenhancing as the local blood-tumor-barrier remains intact [2].

Moreover, these band-like lesions are hyperintense on DWI with low ADC value, which is recognized as a restricted diffusion pattern. Commonly, brain metastases tend to demonstrate facilitated diffusion in the form of elevated ADC values [13]. However, if the primary tumor is lung or breast cancer, it has been shown that brain metastasis with restricted diffusion on DWI is not uncommon [14]. Additionally, brain metastases from lung adenocarcinoma with EGFR mutations tend to have lower ADC value on MRI than metastases with wild EGFR type [15]. Ayzenberg et al. reported a case with LM from lung adenocarcinoma that demonstrated restricted diffusion on DWI, tumor cells of the subarachnoid space, and intracapillary infiltration of tumor cells [7]. Cheng et al. hypothesize that the restricted diffusion surrounding the ventral brainstem is caused by tumor cells concentrating in the cistern around the brainstem and infiltrating into the circumferential perforating arteries along the ventral brainstem surface, resulting in microinfarctions [9].

Our patient had a history of early-stage lung adenocarcinoma with EGFR mutation, which was completely resected by thoracoscopic surgery. PET was not performed, but local recurrence of lung adenocarcinoma and its remote metastases were not revealed with systemic computed tomography using a contrast agent. Brain MRI showed a contrast-enhanced lesion within the left temporal lobe, suggesting parenchymal brain metastases, but tumor cells were not detected in the CSF. Therefore, we could not prove that the band-like lesion surrounding the ventral brainstem was LM from lung adenocarcinoma. However, given the image similarity with previously reported cases and our patient’s history of lung adenocarcinoma with EGFR mutation, we considered that the band-like lesion in our patient was likely to be LM from lung adenocarcinoma. Negative CSF cytology in our patient may be due to inadequate sampling including the number of attempts, sample volume, and time to fixation [7,16,17].

The second important clinical point is that concurrent with identifying the brainstem lesion, our patient showed a rapid cognitive decline, primarily a deficit in object-naming ability. Bartels et al. showed that a high proportion of patients with lung cancer (67.0%) had cognitive impairment, with a similar prevalence between patients with small cell lung cancer (SCLC) and non-small cell lung cancer (NSCLC) [18]. Here, we can assume the following two mechanisms of cognitive decline in our patient. First, LM may have also occurred on the cerebral surface, leading to cognitive decline. Tumor cells on the cerebral subarachnoid space may cause microinfarctions due to intracapillary infiltration via Virchow-Robin spaces, and tumor cells may lead to a metabolic competition of the underlying neuronal tissue, resulting in neuronal dysfunction and subsequent cognitive decline [19,20]. In our patient, the initial and only MRI did not show any findings suggestive of LM of the cerebral surface. However, IMP-SPECT revealed regional cerebral hypoperfusion, including bilateral inferior frontal, orbital, and anterior cingulate gyri. We considered that this hypoperfusion might have reflected LM in the cerebrum that was not detectable on MRI.

A second possible mechanism is that paraneoplastic anti-neuronal antibodies may lead to cognitive decline. Bartels et al. reported that more than one-third of patients with lung cancer had neuronal autoantibodies that were found to be associated with cognitive decline [18]. We examined patient sera using a commercially available paraneoplastic neurological syndrome antibody panel and found no antibodies. However, most of the antibodies in the panel target intracellular antigens and are associated with SCLS. Bartles et al. reported that, among patients with NSCLC, immunoglobin A autoantibodies targeting the N-methyl-D-aspartate receptor, which is a neuronal surface antigen, and autoantibodies against currently unknown antigens are associated with cognitive decline [18]. As we did not probe for these antibodies, we are unable to exclude the possibility that paraneoplastic anti-neuronal antibodies were involved in our patient’s cognitive decline.

## Conclusions

On MRI, we presented a patient with a band-like lesion surrounding the ventral brainstem with T2WI/FLAIR and DWI hyperintensity without gadolinium enhancement. Furthermore, concurrent with identifying the brainstem lesion, our patient showed a rapid cognitive decline. Recent literature reports similar brainstem lesions as a unique LM in patients with lung adenocarcinoma with EGFR gene mutation. Therefore, if MRI shows this brainstem finding, repeated and appropriate CSF cytology is needed to detect tumor cells. Furthermore, if a patient with lung adenocarcinoma shows a cognitive decline, cerebral LM and auto-antibodies primarily targeting neuronal surface antigens should be considered.
